# Return to Full Functioning after Graded Exercise Assessment and Progressive Exercise Treatment of Postconcussion Syndrome

**DOI:** 10.1155/2012/705309

**Published:** 2012-01-16

**Authors:** John G. Baker, Michael S. Freitas, John J. Leddy, Karl F. Kozlowski, Barry S. Willer

**Affiliations:** ^1^Department of Nuclear Medicine, University at Buffalo, SUNY, Buffalo, NY 14214, USA; ^2^Department of Orthopedics, University at Buffalo, SUNY, Buffalo, NY 14221, USA; ^3^Department of Family Medicine, University at Buffalo, SUNY, Buffalo, NY 14215, USA; ^4^Department of Kinesiology, Canisius College, Buffalo, NY 14208, USA; ^5^Department of Psychiatry, University at Buffalo, SUNY, Buffalo, NY 14215, USA

## Abstract

Exercise assessment and aerobic exercise training for postconcussion syndrome (PCS) may reduce concussion-related physiological dysfunction and symptoms by restoring autonomic balance and improving cerebral blood flow autoregulation. In a descriptive pilot study of 91 patients referred to a university clinic for treatment of PCS, a subset of 63 patients were contacted by telephone for assessment of symptoms and return to full daily functioning. Those who experienced symptoms during a graded exercise treadmill test (physiologic PCS, *n* = 40) were compared to those who could exercise to capacity (PCS, *n* = 23). Both groups had been offered progressive exercise rehabilitation. Overall 41 of 57 (72%) who participated in the exercise rehabilitation program returned to full daily functioning. This included 27 of 35 (77%) from the physiologic PCS group, and 14 of 22 (64%) from the PCS group. Only 1 of the 6 patients who declined exercise rehabilitation returned to full functioning. Interpretation of these results is limited by the descriptive nature of the study, the small sample size, and the relatively few patients who declined exercise treatment. Nonetheless, exercise assessment indicates that approximately one third of those examined did not have physiologic PCS.

## 1. Introduction

Concussion is most commonly defined as a trauma-induced alteration of mental status that may or may not involve loss of consciousness [1]. Traumatic brain injury and concussion are leading public health problems in the USA [2]. Rates of traumatic brain injury are highest for young children and men, most often from falls and motor vehicle accidents [2]. Sport-related concussion is also a leading public health problem with an estimate of 1.6 to 3.8 million each year in the United States alone [3]. While 85% of these injuries are considered to be mild, a proportion of them may have long-term effects [4]. There is increasing awareness regarding the personal, medical, and societal costs of concussion, including the reported effects of repeated concussions [5–8]. 

While most patients who suffer a concussion experience resolution over the course of 7–10 days, up to 10% of patients continue to have symptoms [9, 10]. Postconcussion syndrome (PCS) is a constellation of physical, cognitive, and emotional symptoms that persist after concussion [11]. 

Currently, there is no gold standard for diagnosis of PCS. DSM IV [12] and WHO ICD-10 [13] diagnostic criteria for PCS are symptom based and often lack agreement and specificity. Large differences in the prevalence of PCS have been observed when both criteria are applied to the same population [14]. Treatment of PCS has traditionally included rest, education, neurocognitive rehabilitation, and antidepressants, with little evidence of success [11]. 

Concussion management has been moving toward an individualized, patient-centered approach to assessment and treatment, and more athletes and nonathletes are being treated at specialized concussion clinics [10]. Nonetheless, very little outcomes research has been conducted on the effectiveness of treatment for concussion.

As a university concussion clinic, we have been developing a diagnostic and treatment approach for PCS based on a physiological theory of concussion. This approach proposes that a fundamental cause of refractory PCS is persistent physiologic dysfunction [11]. Physiologic dysfunction includes altered autonomic function and impaired autoregulation of cerebral blood flow. One recent study supports the safety and reliability of exercise assessment of PCS [15]. Another study provides support for a program of progressive subsymptom threshold exercise in ameliorating PCS [16]. Exercise assessment and aerobic exercise training may reduce concussion-related physiological dysfunction by restoring autonomic balance and improving cerebral autoregulation of blood flow [17–21]. 

The purpose of this paper is to provide initial descriptive information on the functional outcomes (return to athletic activity, work, or other daily activities) of this assessment and treatment approach to PCS. This study makes use of available clinic data and followup phone interviews, and it addresses the question of whether there is support for future controlled studies of this diagnostic and treatment approach to PCS. 

## 2. Materials and Methods

### 2.1. Patients

A retrospective chart review of clinical information was performed for patients who returned for exercise assessment of PCS at the University at Buffalo Sports Medicine Concussion Clinic between 2007 and 2009. A total of 91 cases were included in this sample, based on returning to the clinic for a graded exercise assessment. A subset of 63 cases were available for followup telephone assessment of residual symptoms and return to full daily functioning. The study was approved by the Institutional Review Board at the University at Buffalo.

### 2.2. Acceptance Criteria

Patients who experienced more than 3 persistent symptoms at rest for a period of more than 3 weeks as a result of a concussion were candidates for exercise assessment of PCS. Individuals determined to have a physiological basis for PCS, based on exacerbation of symptoms during exercise testing, are referred to as the physiological PCS group (P-PCS). They were compared to individuals who were able to exercise to maximum capacity without symptom exacerbation. This later group, whose PCS symptoms may be related to other causes, are referred to as the PCS group. 

All of the patients in both the P-PCS and PCS groups were offered a graduated exercise rehabilitation program. A small proportion of individuals in both the P-PCS and PCS groups declined this exercise rehabilitation program. 

### 2.3. Assessment

A prospective telephone survey of patients who returned for exercise assessment, and who were able to be reached by phone for followup, was performed at an average of two years following injury (range 4 to 73 months). Based on patients' description during this phone interview they were categorized as fully recovered (returned to sports, work, school, etc., as desired), partially recovered (returned to most activities but perhaps unable to play sports or some other desired activity), or not recovered (unable to perform usual activities). The partial recovery and not recovered categories were combined for the analyses. Symptoms at initial exercise assessment and at followup were recorded as present or absent. Symptoms were considered to be physical: headache, dizziness/poor balance; affective: sad or depressed mood, irritability, insomnia, fatigue; or cognitive: concentration or memory difficulties, based on a confirmatory factor analysis of the graded symptom checklist [22].

### 2.4. Groups

Of the 91 patients who returned for exercise assessment, 65 experienced symptoms during the test prior to maximum exercise activity and thus met criteria for physiologically based PCS (P-PCS). The remaining 26 patients were able to exercise to maximum capacity (PCS). Thus, 71% of cases (65/91) were assessed as having a physiological basis for their PCS symptoms (P-PCS), while the remaining cases were considered to be experiencing PCS symptoms related to other causes (PCS).

Unfortunately, the P-PCS and PCS groups are not of equal size for analysis purposes. The proportion of each of the groups who could be reached by telephone for followup is also different (40 of 65 or 62% versus 23 of 26 or 88%). The patients available for followup (40 + 23 = 63) were compared to those unavailable for followup (25 + 3 = 28) on demographic, time interval, and symptom variables. Nonetheless, it is unknown whether there were any other differences that could introduce a systematic bias when comparing patients in the P-PCS group to those in the PCS group. There are random missing values in the results for the patients included in both groups. These missing values are indicated by a superscript in the tables. 

The PCS group was classified according to the primary diagnosis for their persistent symptoms. These primary diagnosis categories included cervicogenic etiology, migraine, depression, posttraumatic stress disorder, and residual visual symptoms. 

### 2.5. Statistics

Group comparisons for categorical variables employed the chi-square test, or the Fischer's exact test for small samples. Independent sample *t*-tests were used for continuous variables and allowed for comparison of unequal size groups. After tabulating descriptive statistics, a step-wise logistic regression analysis was performed to determine the relative association of demographic and symptom variables at initial assessment with functional outcomes at followup. A significance level of *P* < 0.05 was used throughout the statistical comparisons, and in the step-wise logistic regression analysis. 

## 3. Results and Discussion

### 3.1. Results

**Figure 1:**
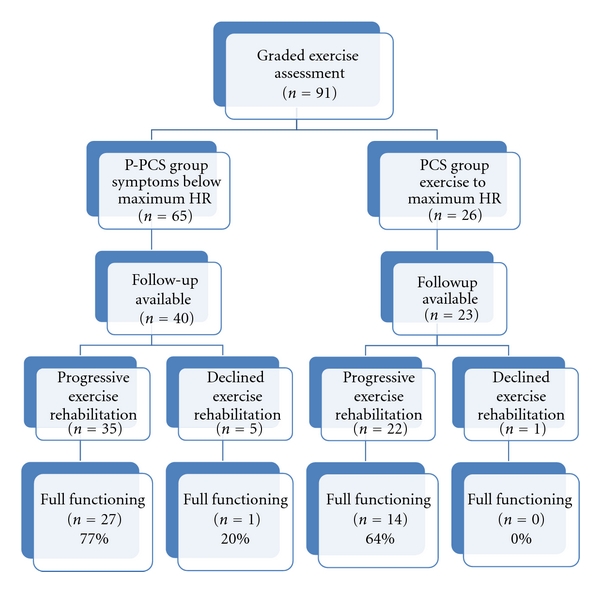
Graded exercise assessment and progressive exercise treatment for P-PCS and PCS groups.

A flow chart is presented in Figure 1 that describes graded exercise assessment, progressive exercise treatment, and full daily functioning at followup for the P-PCS and PCS groups included in this sample. The first two rows of the flow chart include all 91 patients, regardless of whether followup information was available. The remaining three rows include only the 63 patients who were available for followup. The fourth row describes participation in the exercise rehabilitation program, and the fifth row describes return to full daily functioning at followup assessment. 

### 3.2. All Patients (*n* = 91)

A comparison of all the P-PCS and PCS patients, with and without followup information, is presented in Table 1. As can be seen in Table 1, there were no significant differences between the P-PCS and PCS groups for age, gender, or time from injury to assessment. A relatively higher percentage of patients in the P-PCS group endorsed each of the individual symptoms at the time of initial exercise assessment, with the exception of sad or depressed mood. This difference reached statistical significance for headache and fatigue. The primary diagnosis for patients in the PCS group (*n* = 23 out of 26), who were thought to have other than physiologic causes for PCS, included cervicogenic etiology (52%), migraine headache (18%), depression (22%), posttraumatic stress disorder (4%), and residual visual symptoms (4%). 

### 3.3. Patients with (*n* = 63) and without (*n* = 28) Followup Information

Table 2 compares patients with and without followup information. Followup information was available for 63 of the 91 patients (69%) who returned for exercise assessment. As seen in Table 2, the 63 patients with followup information did not differ from the 28 patients without followup information on age, gender, time from injury to the exercise test, or the individual symptoms they endorsed at the initial exercise assessment. There was a longer interval between injury and attempted followup for the 28 patients without followup information (*M* = 39.4 months, SD = 15.3, three missing values) compared to the 63 patients with followup information (*M* = 23.5 months, SD = 13.4, two missing values), *P* < 0.000.

### 3.4. Patients with Followup Information (*n* = 63)

Table 3 presents information on endorsement of individual symptoms at followup for patients in the P-PCS and PCS groups. As can be seen in Table 3, there were no significant differences between the groups at followup. 

Referring back again to Figure 1, and looking at the fifth row, the percentage of patients in the P-PCS group who participated in exercise treatment and returned to full daily functioning (77%) is somewhat greater than the percentage of patients who returned to full daily functioning in the PCS group (64%). This difference did not reach statistical significance, *P* < 0.37. The total number of patients shown in the fifth row of Figure 1 who returned to full daily functioning with exercise rehabilitation is 41 of 57 or 72%. Of the 6 patients in the fifth row of Figure 1 who declined exercise treatment (5 in the P-PCS group and 1 in the PCS group), only 1 returned to full daily functioning. 

Looking at the fourth and fifth rows of Figure 1, within the P-PCS group on the left side (*n* = 40), the proportion of patients who returned to full functioning was greater for those who completed the exercise treatment (27 of 35 returned to full functioning) compared to those who did not complete the exercise treatment (1 of 5 returned to full functioning). This difference reached significance using a Fisher's exact test, *P* < 0.02. The proportion of patients who returned to full daily functioning was also greater when all those who exercised (41 of 57, columns 1 and 3) were compared to all those who did not exercise (1 of 6, columns 2 and 4). This difference also reached significance using a Fischer's exact test, *P* < 0.02. Much caution is needed in interpreting these statistical comparisons due to the small number of patients who did not exercise. 

### 3.5. Regression Analysis

A stepwise logistic regression analysis included return to full daily functioning versus disability or partial return as the dichotomous-dependent variable. A total of 61 of the 63 patients had complete data for all of the variables used in the logistic regression equation. The independent or predictor variables included time from injury to followup, age, gender, time from injury to the exercise test, results of the exercise test, participation in exercise treatment, and initial symptoms. The time in months to followup was entered first as a separate block to control for time since injury. Then, the relative associations of the other independent variables were compared to determine which predictor variable to enter next. Age showed the strongest significant association with return to full daily functioning at followup and was thus entered into the model. 

Table 4 presents the relative associations of the remaining variables after time from injury to followup and age were entered into the model. As can be seen in Table 4, symptom report of irritability showed the next strongest association with return to full daily functioning, followed by concentration/memory difficulties, time from injury to the exercise assessment, insomnia, participation in exercise treatment, performance on the exercise assessment, and then the remaining symptoms. Once time since injury and age were entered into the model, none of the remaining predictor variables reached significance, although irritability approached significance (*P* < 0.06). The model thus contained two independent variables (time since injury and age). This is less than 6 variables, the maximum number that could be entered with 61 participants and still provide adequate statistical power. Unfortunately, the total number of participants who did not participate in the exercise treatment was only six, and so there may not have been adequate statisitical power for this variable to reach significance. 

### 3.6. Discussion

These descriptive comparisons and the results of the logistic regression equation provide information on this pilot sample of individuals referred to a university concussion clinic for assessment and treatment after concussion. This sample is somewhat unique in that patients with persistent symptoms after concussion were invited to return for exercise assessment and treatment of their symptoms. Of course, inferences made from these results need to be interpreted cautiously, due to the small sample size. 

The patients who returned were assessed for the ability to exercise to maximum capacity without experiencing symptoms according to a structured treadmill protocol developed at the concussion clinic. A little more than two thirds experienced symptom exacerbation during exercise, and more of these patients had endorsed headache and fatigue symptoms at rest prior to the exercise test. These patients (P-PCS group) were offered a structured exercise treatment program, and followup data were available for many of the patients in this group. The exercise treatment program was based on the heart rate at which they experienced symptoms. The group of patients who could exercise to maximum capacity (PCS group) was also offered a structured exercise program, and followup data was available for most of these patients. The followup data showed that about two thirds of the patients with followup information who were referred to the clinic for persistent symptoms after concussion returned to full daily functioning. It is difficult to assess the effects of the exercise rehabilitation program on return to full daily functioning, since the number of patients who did not participate is small. 

The logistic regression analysis identified younger age as the strongest significant predictor of return to full daily functioning for patients from both the P-PCS and PCS groups. Report of irritability, an affective symptom, at initial assessment approached significance. Whether a patient could exercise to maximum capacity without experiencing symptoms at initial exercise testing did not significantly predict return to full daily functioning. We interpret this to reflect the fact that almost all of those who failed or passed the exercise test were treated with controlled exercise rehabilitation. Whether a patient participated in the exercise rehabilitation program also was not significantly associated with return to full daily functioning. Since only six patients declined the exercise program, this result could reflect low statistical power.

The results of a previous study conducted in the same university concussion clinic [16] suggested that exercise treatment for PCS is beneficial if the exercise program is individualized, if its progression is controlled in a quantitative manner, and provided that it is administered at the appropriate time after concussion. This previous study suggested that some patients with PCS have a persistent physiological disequilibrium and that controlled aerobic exercise training assists in the recovery of physiological homeostasis. It was proposed in this previous study that symptom-limited exercise testing and progressive subsymptom threshold aerobic exercise training are safe and, as opposed to treatments that modify symptoms (e.g., pain or antidepressant medications), address a fundamental physiological dysfunction in some patients with PCS. It was concluded that, given that there is evidence of physiological dysfunction in concussion and in PCS, physiological assessment should be studied further for a potential role in the diagnosis of concussion and PCS and for helping to determine when patients are ready to resume school, work, and athletic activities.

With respect to functional outcomes of treatment for postconcussion syndrome, as noted in a recent review paper [23], there are three randomized controlled trials that used education, support/reassurance, coping strategies, information sheets on gradual return to normal activities, ongoing advice, and regular followup visits. Wade et al. [24] found that this approach improved daily social functioning and reduced PCS symptoms at 6 months in adults, but others found that education and early treatment inadvertently enhanced patients' consciousness of their symptoms and increased disability [25, 26]. Thus, results of this form of treatment are mixed. A recent systematic review of psychological interventions for the treatment of PCS included cognitive behavioral therapy or CBT. Three randomized controlled trials and seven other studies of CBT all found some benefit, although there were limitations in study design [27]. 

To our knowledge, other studies that look at return to full daily functioning after treatment for PCS are somewhat limited in number. The descriptive results of the present pilot study suggest some hypotheses for future controlled studies of graded exercise assessment and progressive exercise treatment for patients who are experiencing persistent symptoms after concussion. Older age may be a consideration for response to treatment and may be an indication for alterations in treatment protocols to maximize benefit, for both patients who can and who cannot exercise to maximum capacity without experiencing symptoms. The small number of patients with P-PCS who did not elect exercise treatment did not for the most part return to full daily function when compared to those who completed exercise treatment. This also suggests the need to study the basic mechanisms behind the response to controlled exercise rehabilitation for patients who exhibit physiologic dysfunction on an exercise test after concussion. 

This presentation of pilot results is limited by the relatively small sample size, and by only including a subsample of participants with followup information. Participants without followup information generally were those who did not return to the clinic for followup, or those who could not be contacted by phone. There could be differences between this group and the group who did have followup information. Initial comparisons suggested that the two groups did not differ in terms of demographics and other available variables. The small sample size and descriptive nature of the study also limit the inferences that can be made from the results of the statistical comparisons and the regression analysis. Nonetheless, a rate of return to full daily functioning at an average of two years after injury of almost three quarters of PCS patients who participated in exercise rehabilitation would appear to indicate the need for further study.

## 4. Conclusion

The descriptive results of the present pilot study indicate the usefulness of monitoring outcomes, including return to full daily functioning, for assessment and treatment of concussion and postconcussion syndrome. As multidimensional assessment and treatment of postconcussion syndrome is further developed in concussion clinics, the effectiveness of various treatment approaches can be monitored and reported in the literature. This initial descriptive information revealed exercise assessment is useful in identifying those with physiologic PCS. The diagnosis of those without physiologic PCS included cervicalgia, migraine, ocular-vestibular dysfunction, and emotional disturbance. This study suggests that PCS represents more than one disorder and perhaps PCS is more appropriately described as postconcussion disorders. 

## Figures and Tables

**Table 1:** All patients in the P-PCS (could not exercise to maximum capacity) and PCS (could exercise to maximum capacity) groups.

		P-PCS	PCS	
		*n* = 65	*n* = 26	Significance
Age (years)	Mean (SD)	28.9^a^ (14.0)	27.0^b^ (12.9)	*P* < 0.56
	Range	13–54	15–58	
Gender	Male (%)	33 (51%)	14 (54%)	*P* < 0.82
	Female (%)	32 (49%)	12 (46%)	
Time from injury to assessment (months)	Mean (SD)	7.6^a^ (10.3)	11.7^b^ (18.0)	*P* < 0.19
	Range	1–54	1–71	

Percent endorsing individual symptoms at initial exercise assessment				

Headache*		97%	81%	*P* < 0.02
Dizziness/Balance		79%	65%	*P* < 0.29
Sad/Depressed		49%	62%	*P* < 0.36
Irritability		62%	54%	*P* < 0.64
Fatigue*		86%	58%	*P* < 0.01
Insomnia		51%	46%	*P* < 0.82
Concentration/Memory		83%	65%	*P* < 0.09

^a^Three cases with a missing value. ^b^One case with a missing value. *Significant at *P* < 0.05.

**Table 2:** Comparison of patients with followup information (*n* = 63) to patients without followup information (*n* = 28).

		With followup	Without followup	Significance
Total *n* = 91		*n* = 63	*n* = 28	
Age (years)	Mean (SD)	27.4^a^ (13.1)	30.6^b^ (14.9)	*P* < 0.33
	Range	12–58	13–68	
Gender	Male (%)	34 (54%)	13 (46%)	*P* < 0.51
	Female (%)	29 (46%)	15 (54%)	
Time to assessment (months)	Mean (SD)	7.5^a^ (12.4)	11.9^b^ (14.2)	*P* < 0.16
	Range	1–71	1–60	

Percent endorsing individual symptoms at initial exercise assessment				

Headache		89%	100%	*P* < 0.10
Dizziness/Balance		79%	64%	*P* < 0.19
Sad/Depressed		57%	43%	*P* < 0.26
Irritability		56%	68%	*P* < 0.36
Fatigue		78%	79%	*P* < 1.00
Insomnia		52%	43%	*P* < 0.50
Concentration/Memory		76%	82%	*P* < 0.60

^a^One case with a missing value. ^b^Three cases with a missing value.

**Table 3:** Percent of patients with followup information (*n* = 63) in P-PCS and PCS groups who endorsed individual symptoms.

	P-PCS	PCS	
	(*n* = 40)	(*n*=21)^a^	Significance
Headache	33%	38%	*P* < 0.78
Photophobia	08%	14%	*P* < 0.41
Dizziness/Balance	18%	19%	*P* < 1.00
Sad/Depressed	07%	14%	*P* < 0.40
Irritability	20%	14%	*P* < 0.73
Fatigue	26%^b^	19%	*P* < 0.75
Insomnia	15%	14%	*P* < 1.00
Concentration/Memory	23%	14%	*P* < 1.00

^a^Two cases with missing values. ^b^One case with a missing value.

**Table 4:** Relative associations of predictor variables with return to full daily functioning^a^.

Predictor variables	Relative association with return to full daily functioning	
Variables in model	Wald statistic	Significance
Time to f/u	0.52	*P* < 0.47
Age*	8.15	*P* < 0.004

Variables not in model	Score statistic	Significance

Irritability	3.44	*P* < 0.06
Concentration	2.70	*P* < 0.10
Time to test	2.38	*P* < 0.12
Insomnia	2.35	*P* < 0.13
Exercise treatment	2.26	*P* < 0.14
Test result	1.08	*P* < 0.30
Fatigue	0.44	*P* < 0.51
Sad/Depressed	0.36	*P* < 0.55
Gender	0.13	*P* < 0.72
Headache	0.02	*P* < 0.89

^a^Two cases with missing values. *Significant at *P* < 0.05, f/u = followup.
